# A scoping review of sense of coherence and salutogenesis among LGBTQ+ populations

**DOI:** 10.1093/heapro/daaf049

**Published:** 2025-04-23

**Authors:** Caroline E Brett, Hannah Madden, Darci Tillbrook, Vivian D Hope

**Affiliations:** School of Health in Social Science, The University of Edinburgh, Old Medical School, Teviot Place, Edinburgh EH8 9AG, United Kingdom; Public Health Institute and School of Public and Allied Health, Liverpool John Moores University, 79 Tithebarn Street, Liverpool L2 2ER, United Kingdom; Public Health Institute and School of Public and Allied Health, Liverpool John Moores University, 79 Tithebarn Street, Liverpool L2 2ER, United Kingdom; Public Health Institute and School of Public and Allied Health, Liverpool John Moores University, 79 Tithebarn Street, Liverpool L2 2ER, United Kingdom

**Keywords:** sense of coherence, salutogenesis, LGBTQ+ populations, positive psychology

## Abstract

Salutogenesis and sense of coherence can buffer the effects of external threats such as stigma, discrimination, and emergencies. Lesbian, gay, bisexual, trans, and queer (LGBTQ+) communities globally face discrimination, prejudice, and victimization. Understanding salutogenesis and sense of coherence among LGBTQ+ people could improve policy and health promotion responses and reduce the impact of these stressors on LGBTQ+ people’s health. This scoping review addressed the following question: To what extent has salutogenesis, either qualitatively or through measurement of sense of coherence, been explored among LGBTQ+ populations globally? Systematic searches of five databases identified 448 unique sources: 413 were excluded through title and abstract screening, and 18 by full-text screening. Of the 17 included studies, 14 were quantitative, two qualitative, and one mixed methods. Studies recruited participants from 11 high-income countries. Two-thirds (11) recruited participants only from within the LGBTQ+ community and six had more broadly based samples. The studies sampled a diverse range of subpopulations and subgroups from a variety of contexts limiting generalizability. The quantitative measure of sense of coherence most often used was the SoC-13 (*n* = 7), however, heterogeneity in the results reporting prevented quantitative synthesis. The limited evidence suggests that sense of coherence may be lower in LGBTQ+ populations than in comparison groups of cisgender heterosexuals, at least in some contexts, and is related to measures of wellbeing and discrimination. Further research is needed to understand how sense of coherence and its dimensions influence, and is influenced by, LGBTQ + people’s life experiences and resources and their responses to external stressors.

**Contribution to Health Promotion**

- Salutogenesis and sense of coherence can buffer the effects of external threats, such as the discrimination experienced by minority groups. It could also buffer the impacts of public health emergencies, such as pandemics.
- Our review found that in lesbian, gay, bisexual, trans, and queer (LGBTQ+) populations, sense of coherence is related to wellbeing and discrimination, and more limited evidence suggests sense of coherence may be lower compared to cisgender heterosexuals.
- Therefore, the three dimensions of sense of coherence—particularly the behavioural aspect of manageability—should be considered when developing and implementing health promotion interventions to improve health and wellbeing in the LGBTQ+ community.

## INTRODUCTION

The salutogenic approach to health, first proposed by Aaron Antonovsky (Antonovsky 1979, 1987), focusses on the factors and resources that enable people to maintain their health and wellbeing in the face of adversity. Sense of coherence is a core concept in salutogenesis that Antonovsky proposed to comprise of three dimensions. *Comprehensibility*—the cognitive dimension—refers to the perception that the circumstances of one’s life are structured, predictable, and explicable. *Manageability*—the behavioural dimension—captures the perception that the resources available to a person are enough to meet the demands posed by life circumstances. *Meaningfulness*—the motivational dimension—refers to the perception that tackling these demands is worthwhile and meaningful for the individual (Antonovsky 1979, 1987, Mittelmark et al. 2022). Many researchers posit sense of coherence as health-promoting (Antonovsky 1996), enabling tension management and successful coping by mobilizing what Antonovsky terms ‘generalized resistance resources’, such as social support, self-efficacy, personality, and culture (Antonovsky 1979, 1987).

Sense of coherence is generally measured using Antonovsky’s original 29-item sense of coherence scale (abbreviated to SoC-29) or a 13-item version (SoC-13) of this scale (Naaldenberg et al. 2011), although shorter versions of the scale have been developed, such as, the SoC-9 (Klepp et al. 2007, Eriksson and Mittelmark 2017). All of the above scales yield scores on the three dimensions of comprehensibility, manageability, and meaningfulness, and have been shown to have good validity, reliability, and consistency in a variety of populations (Klepp et al. 2007, Naaldenberg et al. 2011, Eriksson and Mittelmark 2017). Sense of coherence has been shown to be positively associated with mental and physical health (Eriksson and Lindström 2006, 2007), as a mediator between resources and subjective health and wellbeing (Eriksson and Mittelmark 2017), and as a buffer for the impacts of public health emergencies, such as the COVID-19 pandemic, on stress and mental health (Schäfer et al. 2020).

Lesbian, gay, bisexual, trans, and queer (LGBTQ+) communities face varying degrees of discrimination, prejudice, and victimization across the world (Katz-Wise and Hyde 2012, Pachankis and Branstrom 2018). In some countries, discriminative legislation has been repealed, with a marked increase in legal protections for LGBTQ+ people in recent decades (Herre and Arriagada 2024). However, other countries have seen increased criminalization of LGBTQ+ people in recent years (Herre and Arriagada 2024). Even in countries with legislation protecting LGBTQ+ people against discrimination, this remains a problem, and in some countries, this may be increasing (Poushter and Kent 2021). For example, in the USA there have been homophobic protests at drag shows and increasing visibility of anti-trans lobby groups (United Nations 2022, Gabbatt et al. 2023). Changes in governments can result in marked changes in policy, as happened following the inauguration of the 47^th^ President of the USA in January 2025. The incoming administration rapidly implemented a number of executive orders that impacted LGBTQ+ populations, particularly those who are trans and non-binary. These were informed by ‘Project 2025’ a legislative programme propagated by a right-wing ‘think tank’ (The Heritage Foundation 2024). Public health organizations have highlighted that the aims of Project 2025 are a threat to public health and, in particular, the health of gender and sexual minorities in the USA (American Public Health Association 2025). For example, executive orders issued in January 2025 instructed the US Centers for Disease Control to remove references to gender and trans identities from their health information, websites, and academic publications, which will have negative impacts on the well-being of LGBTQ+ communities (Clark and Abbasi 2025). In the UK there are indications that hate crimes against LGBTQ+ people, particularly incidents against trans individuals, have increased in recent years (Home Office 2023), whilst the British Social Attitudes survey has found that the proportion of people saying that same-sex relations are ‘not wrong at all’ has plateaued since 2017 after gradual but consistent rise over the preceding two decades (Clery 2023). The most recent British Social Attitudes survey also found that attitudes towards transgender people have become less liberal over the last three years; for example, the proportion agreeing that the sex recorded on birth certificates should be changed if someone transitioned has decreased from 53% in 2019 to just 30% in 2023 (Clery 2023). These changes have been accompanied by a rise in anti-LGBTQ+ rhetoric, with the alternative right-wing media and the so-called ‘culture wars’ challenging the equality rights of LGBTQ+ communities (Castle 2019, Siegel 2020).

LGBTQ+ communities experience multiple health and wellbeing inequalities, with these inequalities reflecting the negative impact of the discrimination, prejudice, and marginalization that they experience (King et al. 2008, Kelleher 2009, Hendricks and Testa 2012, Frost et al. 2015, Borgogna et al. 2019). These health inequalities include disparities in physical health (Bränström et al. 2016, Jackson et al. 2016, Simoni et al. 2017, Tran et al. 2023), higher levels of smoking and alcohol and drug use (Jackson et al. 2016, Rosner et al. 2021) and sexual health (Sullivan et al. 2021). Research across many geographic regions has consistently shown LGBTQ+ people experience poorer mental wellbeing than cis-gendered heterosexuals, including higher rates of anxiety, depression, suicide ideation and attempts; and elevated risks for depression (King et al. 2008, Plöderl and Tremblay 2015, Zeeman et al. 2019).

In addition, LGBTQ+ persons experience widespread barriers to accessing healthcare, and report negative experiences of health services; including stigma, denial or refusal of care, and physical and verbal abuse (Elliott et al. 2015, Ayhan et al. 2020). There are also health disparities in the LGBTQ+ community; bisexual and trans individuals are at even higher risk of physical and mental ill health than their cisgendered or gay/lesbian counterparts (Dilley et al. 2010, Colledge et al. 2015, Booker et al. 2017).

The recent Coronavirus disease 2019 (COVID-19) pandemic may have had a disproportionate impact on the wellbeing of LGBTQ+ communities (Flentje et al. 2020, Gonzales et al. 2020, Sanchez et al. 2020, Santos et al. 2021, Hope et al. 2023), potentially amplifying the existing health inequalities. For example, evidence from studies conducted in the USA showed LGBTQ+ persons were at higher risk of contracting COVID-19, and experienced worse mental health and more problem drinking during the pandemic (Akré et al. 2021, Sell and Krims 2021). A UK study found that LGBTQ+ people, when compared to the rest of the general population, were significantly more likely to report being unable to access sufficient food and required medication, and were more likely to be eating less healthily, exercising less regularly, experiencing poorer quality sleep and taking more pain medicine than usual (Hope et al. 2023). Isolation and being unable to access social networks were concerns commonly reported by LGBTQ+ participants in this study (Hope et al. 2023). More recently the LGBTQ+ community was disproportionately impacted by the global outbreak of Mpox during 2022 and 2023 (Vaughan et al. 2022). The response to this may have amplified the stigma experienced by LGBTQ+ community and impacted engagement in healthcare (Bragazzi et al. 2023, Biesty et al. 2024).

A strong sense of coherence may mediate the harms arising from external threats, such as discrimination and stigma (Ying et al. 1997, Lam 2007, Switaj et al. 2013, Baron-Epel et al. 2017, Grothe et al. 2022). For example, sense of coherence has been reported to have a role in mediating the relationship between perceived racial discrimination and both depression and anxiety (Lam 2007), and to be a significant predictor of lower levels of stigma in people with mental illness (Switaj et al. 2013). At the same time, sense of coherence is widely thought to itself be shaped by stressors and life experiences, with stressors such as discrimination and stigma either improving or diminishing the sense of coherence depending on the availability and use of generalized resistance resources and specific life situations (Global Working Group on Salutogenesis 2024).

The three dimensions of sense of coherence—comprehensibility, manageability, and meaningfulness—may be particularly relevant for LGBTQ+ people for whom their LGBTQ+ identity is a source of meaning but whose lifestyles may differ from prevalent heteronormative values, and whose manageability may be impacted by a lack of familial or societal support. Sense of coherence could thus potentially buffer LGBTQ+ individuals from the harmful effects arising from external threats, such as stigma, and the amplification of these during public health emergencies, such as pandemics, while also being improved or diminished by their life experiences.

A better understanding of salutogenesis and sense of coherence among LGBTQ+ people, therefore, has the potential to inform the development of responses to mitigate the harmful impacts of external threats and promote good health and wellbeing in this population that often experiences discrimination, prejudice, and victimization.

The current scoping review aimed to broadly assess the extent to which salutogenesis and sense of coherence have been explored, either qualitatively or quantitatively, among LGBTQ+ communities globally. The secondary aims of this study were to identify the associations between sense of coherence and stigma, discrimination, and mental and physical health, and to highlight gaps in current knowledge.

## METHODS

This scoping review followed a review plan that was prepared prior to commencing the searches (Supplementary Annex 1). A scoping review was undertaken as the focus of this review was to map the body of literature on salutogenesis and sense of coherence in LGBTQ+ populations, and because initial exploratory searches had indicated a heterogeneous and limited body of extant literature.

The review plan was formulated using the Population, Concept, and Context (PCC) framework, and was informed by Arksey and O’Malley’s framework for scoping reviews (Arksey and O’Malley 2005) alongside methodological guidelines for scoping reviews (Peters et al. 2015, 2020). Reporting of the review follows the PRISMA Extension for Scoping Reviews checklist (Tricco et al. 2018) (Supplementary Annex 2).

Ethical approval was not needed as this study is a review of the extant literature, and so did not involve the collection of any new primary data.

### Review question

To what extent has salutogenesis, either qualitatively or through measurement of sense of coherence, been explored among LGBTQ+ populations globally?

### Inclusion and exclusion criteria

The inclusion and exclusion criteria used for the record section are set out in Table 1. The only limits applied were peer-reviewed manuscripts reporting on primary research and published in English. The outcomes of interest were either a quantitative measurement of sense of coherence (e.g. using standardized measures such as SoC-29 or SoC-13 scales) or a qualitative analysis exploring salutogenesis or sense of coherence.

**Table 1.** The inclusion and exclusion criteria

	Inclusion	Exclusion
**PCC components**		
***Population***	Lesbian, gay, bisexual, trans, queer (LGBTQ+) people and communities, and associated terms (such as gender minorities, MSM, etc), including studies of component populations, and where part of a general population study the data is reported separately for LGBTQ+ people	Where data is not reported for LGBTQ + people, or any of the subgroups within this umbrella category.
	Adults (all, or mostly, aged 18 years and over)	Only people aged under 18 years.
***Concept***	Quantitative measure of sense of coherence OR qualitative assessment of salutogenesis or sense of coherence	No quantitative measure of sense of coherence OR qualitative assessment of salutogenesis or sense of coherence
***Context***	All countries	–
**Limits**		
***Language***	In English	Languages other than English
***Study types***	Primary studies: quantitative, qualitative, or mixed methods.	Reviews
		Case reports and series
		Conference abstracts
		Thesis
		Commentaries and editorials

### Search strategy

Searches were conducted on five electronic databases: Medline All, PsycINFO, CINAHL Plus (via EBSCOHost), Web of Science Core Content, and Scopus. The searches were undertaken on 16 February 2023 and then updated on 5 June 2024. The search terms for each of the components of the PCC were first built into strings using the Boolean operator ‘OR’. The searches using these three strings were run in each of the databases, and these searches were then combined using the Boolean operator ‘AND’ to identify sources of interest in each database. The search strategy used in Medline, from which the other database searches were developed, is shown in Supplementary Annex 3.

### Screening

After de-duplication in Endnote, using the Endnote duplicate finder and then by undertaking manual checks by sorting on article title and the name of the first author, the records were then imported into Rayyan (https://www.rayyan.ai/) for screening (undertaken by VH, DT, CB, and HM). The title and abstract screening was undertaken independently by two people, with any disagreements resolved by a third member of the study team.

Full texts were then obtained for all of the sources that were not excluded during the title and abstract screening stage. Full-text screening was then undertaken using the same approach as used for title and abstract screening, but with any disagreement resolved by discussion involving all four of the authors involved in the screening process.

### Data charting

A Microsoft Excel spreadsheet was used to create a form for the extraction and charting of the data. The extraction focussed on key study characteristics (e.g. population, recruitment approach, data collection method, etc.), year of data collection (to account for changes in legislation, understanding, and acceptance that might affect LGBTQ+ people), the measurement of sense of coherence and/or assessment of salutogenesis used (e.g. quantitative measure of sense of coherence used, or qualitative approach taken), the types of analyses undertaken, and the outcomes (e.g. sense of coherence values, and associations between sense of coherence or salutogenesis and health and wellbeing measures).

Data extraction for the included papers was undertaken by the four authors (VH, DT, CB, and HM). One author would extract the data, and then this extraction was reviewed by a second author. To ensure consistency across the four authors’ data extraction, the papers extracted by one author were allocated across the other authors for reviewing.

### Collating, summarizing, and reporting the results

As this was a scoping review, and considering a range of study types, no quality appraisal of the included studies was undertaken. The aim of this review was to summarize existing evidence on the topic to inform future research, and so a descriptive narrative approach was used to synthesize and summarize the charted data. The focus was on how sense of coherence or salutogenesis was explored or measured, what was found, comparisons between different sexual orientation and gender identity groups (e.g. gay and bisexual men vs. heterosexual men, or trans women vs. cisgender women), and any associations that were found with measures of discrimination or stigma, and measures of physical or mental health.

## RESULTS

**Figure 1.**
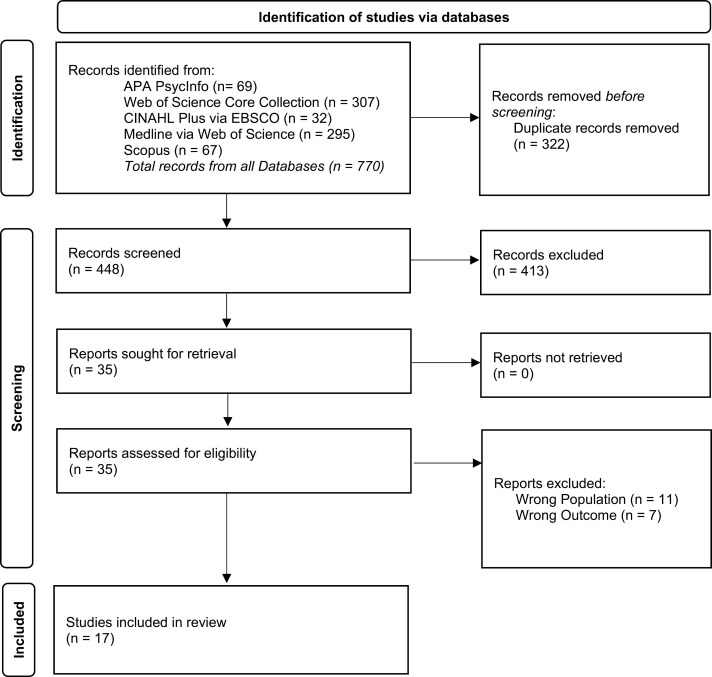
PRISMA flowchart.

The database searches, after de-duplication, yielded a total of 448 unique sources. Of these, 413 were excluded during the title and abstract screening, with the full texts obtained for the remaining 35 sources. The full-text screening resulted in a further 18 of the sources being excluded. The remaining 17 sources were then charted (see Fig. 1).

### Study characteristics

Table 2 summarizes the characteristics of the studies reported in 17 included sources. These studies recruited participants from 11 countries, however, the country of data collection was not explicitly stated in three of the papers. The USA was the most common country of data collection (five studies). The included studies were from North America, Europe, Israel, and Australia; there were no studies from low- or middle-income countries. The year of data collection was given for 12 of the studies, with this ranging from 2007 to 2021.

**Table 2.** Characteristics of the studies report in the 17 included sources

Author/s	Paper title	Publication year	Country	Year of data collection	Population/subgroups	Setting and recruitment	Data collection	Approach
Bjorkman and Malterud	Lesbian women coping with challenges of minority stress: a qualitative study	2012	Norway	2007	Lesbian women.	Recruitment through gay/lesbian websites (incl. those of organisations), and media promotion (gay/lesbian as well as general).	Online survey.	Qualitative
Breidenstein et al.	Psychosocial resources and quality of life in transgender women following gender-affirming surgery	2019	Germany	2016	Trans women who had received gender-affirming surgery aged 18 years and over.	Clinical database search at one Urology Clinic to identify trans women who had received gender-affirming surgery. Those eligible were invited to participate via mail.	Postal paper survey.	Quantitative
Ciria-Barreiro et al.	A comparative study of health and well-being among cisgender and binary and non-binary transgender Adolescents in Spain	2021	Spain	2018	Adolescents (aged 15–18 years).	Data from Health Behaviour in School-aged Children study. Multi-stage stratified sampling to select students at Spanish schools.	Survey completed on digital device at school.	Quantitative
Dyer	Refining research on the intersection between sexual orientation, suicide, and religiosity	2022	USA	2011	Students aged under 30 years and with sexual orientation data	Stratified random sample of students at 74 participating colleges and universities, response rate 26%.	Online survey.	Quantitative
Fish, Williamson, and Brown	Disclosure in lesbian, gay and bisexual cancer care: towards a salutogenic healthcare environment	2019	UK	Unclear: 2017/18	Lesbian, gay and bisexual cancer patients.	Oncology departments in five parts of England plus Community support groups.	Semi-structured interviews.	Qualitative
Issler et al.	The relationship between childhood gender nonconformity, aversive childhood experiences, and mental health in heterosexual and non-heterosexual cisgender men: The buffering effect of sense of coherence	2023	Germany	2021	Cisgendered men	Recruitment through online advertising utilizing social media and distribution of flyers across Germany.	Online survey.	Quantitative
Jastrzębska and Błażek	Questioning gender and sexual identity in the context of self-concept clarity, sense of coherence and value system	2022	Poland^†^	NS	Young adults	Recruitment via social media posts.	Online survey.	Quantitative
Kertzner	Self-appraisal of life experience and psychological adjustment in midlife gay men	1999	North America^†^	NS	Middle-aged gay men (aged 40–55 years)	Recruitment through newspaper advertisements, notices placed in newsletters, electronic bulletin board postings, and flyers in bars.	Self-complete survey and semi-structured interview	Mixed Method: Quantitative and Qualitative
King and Smith	Happy, mature, and gay: Intimacy, power, and difficult times in coming out stories	2005	USA	NS	Gay men and lesbians	Recruitment through advertisements in local publications, and using flyers in a gay bookstore and a women’s bar. Postal survey, including free text question on coming out.	Postal paper survey.	Quantitative
Kotowska, Nizio, and Kurpisz	The health behaviour of homosexual and heterosexual women in context of theory of salutogenesis	2020	Poland	NS	Women: women who have sex exclusively or mostly with women, and women who have sex exclusively with men	Recruitment via social media networks across Szczecin.	Paper survey	Quantitative
Lyons, Pitts, and Grierson	Sense of coherence as a protective factor for psychological distress among gay men: a prospective cohort study	2014	Australia	Baseline survey 11/2010–04/2011; follow-up 11/2011–04/2012	Gay-identified men aged 40 years and older.	Recruitment through adverts social media and websites.	Online survey.	Quantitative
Mahon et al.	Risk and Protective Factors for Social Anxiety Among Sexual Minority Individuals	2021	Republic of Ireland	2018	Adults expressing sexual minority identity. Excluded trans and non-binary.	Recruitment through targeted adverts on social media and LGBTQ+ organizations email lists.	Online survey.	Quantitative
McDaid et al.	Informing theoretical development of salutogenic, asset-based health improvement to reduce syndemics among gay, bisexual and other men who have sex with men: empirical evidence from secondary analysis of multi-national, online cross-sectional surveys	2020	Canada and UK	2014–2015, 2016	Gay and Bisexual men and other men who have sex with men	Recruitment through adverts targeting gay and bisexual and other men who have sex with men using geosocial networking apps and websites, plus community groups, social media, and gay media in Canada.	Two online surveys.	Quantitative
McGarty et al.	Mental health, potential minority stressors and resilience: evidence from a cross-sectional survey of gay, bisexual and other men who have sex with men within the Celtic nations	2021	UK and Republic of Ireland	2016	Gay and Bisexual men, or those who sought sex with other men.	Recruitment through adverts men who have sex with men geosocial networking apps and sociosexual websites.	Online survey.	Quantitative
Shechory Bitton and Noach	Psychological factors and the use of psychoactive substances in relation to sexual orientation: a study on Israeli young adults	2022	Israel	2019	Young adults (aged 18–25 years): either Lesbian/Gay or heterosexual.	Research assistants distributed the questionnaire to young adult on university and college campuses.	Paper survey	Quantitative
Szymanski and Chung	Feminist attitudes and coping resources as correlates of lesbian internalized heterosexism	2003	USA	NS	Lesbian and bisexual women aged 18 years or older.	Online recruitment (list servers), ‘coming out’ group, plus via social networks. Participants asked to distribute additional surveys to eligible contacts.	Postal paper survey.	Quantitative
Veldorale-Griffin and Darling	Adaptation to parental gender transition: stress and resilience among transgender parents	2016	North America^†^	2011–2012	Transgender parents.	Recruitment through gay/lesbian websites (incl. those of organisations), and media promotion (gay/lesbian as well as general).	Online survey.	Quantitative

^†^Country not explicitly stated.

Eleven of the studies (65%) recruited participants only from within the LGBTQ+ community, with six studies having more broadly based samples. Four studies had recruited only gay and/or bisexual men (Kertzner 1999, Lyons et al. 2014, McDaid et al. 2020, McGarty et al. 2021), two only lesbian and/or bisexual women (Szymanski and Chung 2003, Bjorkman and Malterud 2012), and two only trans people (Veldorale-Griffin and Darling 2016, Breidenstein et al. 2019); with three having recruited gay and/or bisexual people more generally (King and Noelle 2005, Fish et al. 2019, Mahon et al. 2021). Of the studies with broader samples four were of young adults or adolescents (Ciria-Barreiro et al. 2021, Dyer 2022, Jastrzębska and Błażek 2022, Shechory Bitton and Noach 2022), one had recruited adult men (Issler et al. 2023), and one had recruited adult women (Kotowska et al. 2020).

The studies used a range of methodological approaches: 14 were quantitative, two qualitative, and one used a mixed methods approach (Table 2). The quantitative studies had all administered surveys, and all but one had used a cross-sectional design with no follow-up. A range of approaches were employed to recruit participants, with most studies having used some form of convenience sampling.

Table 3 summarizes the findings of the included studies. The quantitative studies measured sense of coherence using a number of tools: seven studies used the SoC-13 scale (Szymanski and Chung 2003, Lyons et al. 2014, Breidenstein et al. 2019, McDaid et al. 2020, Ciria-Barreiro et al. 2021, Mahon et al. 2021, McGarty et al. 2021), four studies used the SoC-29 scale (King and Noelle 2005, Kotowska et al. 2020, Jastrzębska and Błażek 2022, Shechory Bitton and Noach 2022), and one the SoC-9 scale (Issler et al. 2023). The measure of sense of coherence used in the remaining two quantitative studies and the mixed methods study were not clearly described. The approaches to the qualitative assessment of salutogenesis varied between the two qualitative studies and the mixed-method study.

**Table 3.** Measurement of sense of coherence or assessment of salutogenesis in the included studies

Author/s	Study aim	Population/subgroups	Sample/subgroup size	Demographics	Measure used	Analysis method/s	Value	Findings
Quantitative								
SoC-29								
Jastrzębska and Błażek	‘To compare heterosexual and cisgender people with non-heteronormative and non-cisgender people regarding their attitudes and the way they perceive significant personal values’	Young adults aged 18–30 years.	Total *n* = 337	Mean age 21.41 (SD = 3.14), Women 66%, men 16%, transgender 13%, and who did not identify their gender 4%. 33% heterosexual, 27% bisexual, 15% homosexual, 4% asexual, 20% other or did not identify their sexual orientation.	SoC-29	Pearson’s *r* correlation, *t*-tests	SoC-29 Total score: Cisgender mean = 103.8 (SD = 26.2), Non-Cisgender mean = 90.4 (SD = 21.8). Heteronormative mean = 108.8 (SD = 27.1), Non-Heteronormative mean = 92.6 (SD = 23.0).	SoC-29 significantly higher for cisgender and heteronormative groups (both *P* < .0001). The higher the self-concept clarity, the higher the SoC-29. Cisgender respondents had higher self-concept clarity and SoC-29 and tended to question their sexuality less frequently. Statistically significant differences were found for cisgender vs. non-cisgender respondents in SoC-29 as well as for heteronormative vs. non-heteronormative people.
*** *** King and Smith	To analyse coming out stories for power motive imagery, intimacy, and difficult times and associations with subjective wellbeing and ego development	Gay men and lesbians	*n* = 107 gay men and lesbian women	65 men, 39 women; 3 not reporting, age 18 to 66 years (mean = 36.65 [SD = 9.68]); 87.9% white.	SoC-29	Content analysis of coming out stories. Correlations between measures including story dimensions. Multiple regression with Ego Development and life satisfaction as outcomes.	The items are rated on a scale from 1 (strongly agree) to 5 (strongly disagree): mean = 2.38, SD = 0.40	‘Life satisfaction and SoC-29 were related to a lack of negative affect in the [coming out] story, though these measures of SWB were not associated with telling more positive stories’.
Kotowska, Nizio, and Kurpisz	To identify the differences and similarities in health behaviours and levels of sense of coherence between women who have sex with women (WSW) and women who have sex only with men.	Women (aged 20–50 years): women who have sex exclusively or mostly with women, & women who have sex exclusively with men	Total *n* = 64, women who have sex with women (33) and women who only have sex with men (31) aged between 20–50 years.	Average age 29.5 years (SD = 7.79)	SoC-29 (Polish version)	t-test for independent tests, chi-square test for independence and Fisher’s exact test	Means (SD): women who have sex with women Total SoC-29 = 111.30 (28.04); comprehensibility = 35.97 (9.09); manageability = 38.70 (12.07); meaningfulness = 35.64 (10.87). women who have sex with men Total SOC = 121.84 (21.29); comprehensibility = 41.03 (10.68); manageability = 46.42 (8.65); meaningfulness = 34.39 (8.48).	Significant difference between women who have sex with women and women who have sex with men; SoC-29 higher in women who have sex with men (*P* = .070). women who have sex with women scored lower on comprehensibility (*P* = .045) and manageability (*P* = .005), but no difference between groups on meaningfulness (*P* = .609).
Shechory Bitton and Noach	To examine the association between sexual orientation, psychological distress, sense of coherence (SOC), social support, and alcohol and cannabis use among Israeli young adults.	Young adults (aged 18–25 years): either Lesbian/Gay or heterosexual.	*n* = 496, *n* = 254 identified as heterosexual and *n* = 242 identified as homosexual.	48% female, mean age 23.1 years (SD = 2.48).	SoC-29 (items were rated on 5-point scales)	Z test, chi-square, analyses of covariance, multiple hierarchical regression Chi-square test, correlations, ANCOVA, multiple hierarchical regression	SoC-29 means (SD): homosexuals: males = 3.34 (0.48) and females = 3.43 (0.53); heterosexual males = 3.69 (0.44) females = 3.56 (0.45).	SoC-29 was higher among heterosexual participants than among Lesbian/Gay participants (*P* < .001). SoC-29 was strongly negatively correlated with psychological distress and moderately positively correlated with relationships with parents and friends. SoC was strongly associated with psychological distress in regression model.
* *SoC-13								
Breidenstein et al.	‘The present study aimed to systematically investigate the existence of different psychosocial resources and QOL (quality of life) in trans women following gender-affirming surgery (GAS)’.	Trans women who had received gender-affirming surgery aged 18 years and over.	*n* = 158	Mean age = 49.8 years (SD = 11.1; range 2277 years).	SoC-13	Group effects were analysed using Kruskal–Wallis tests. Post hoc tests were performed with Mann–Whitney tests.	Study group SoC-13 mean = 4.97 (SD 1.11)	Comparison of SoC-13 with control group indicate no difference (*P* = .727); control women of the normative samples reported in the respective test manuals, SoC mean = 5.00 (SD 0.89).
Ciria-Barreiro et al.	The aim of this study was to describe the wellbeing and mental health of Spanish adolescents by gender identity using a nationally representative sample	Adolescents (aged 15–18 years).	*n* = 1,212 15–18-year-old adolescents: 303 transgender and 909 cisgender.	90 were identified as binary transgender adolescents, 213 as non-binary transgender adolescents. Half were rural; 56% were aged 17–18 years	SoC-13 (6-point Likert scale)	Comparing cis vs trans students means, SDs, adjusted residuals, chi-squared, t-tests ad ANOVA	SoC-13: trans adolescents mean = 3.69 (SD = 1.00),	Difference found between Cisgender (mean = 4.25, SD = 1.01), and transgender (mean = 3.69, SD = 1.00) adolescents (*P* ≤ .001). SoC comparing cisgender (mean = 4.25, SD = 0.01) vs binary transgender (mean = 4.03, SD = 0.99) vs non-binary transgender (mean = 3.56, SD = 0.97) was found to be significantly different (*P* < .001). Post hoc tests showed difference between cisgender and non-binary was significant (*P* < .001), but difference between binary and non-binary was not significant (*P* = .046).
Lyons, Pitts, and Grierson	‘This prospective cohort study investigated whether a sense of coherence (SOC), as a form of coping, helps to protect against psychological distress among middle-aged and older gay men’.	Gay-identified men aged 40 years and older.	*n* = 1,179, at base line and *n* = 372 at follow-up (33% of baseline at follow-up could matched to a baseline survey).	Mean age = 49.3 years; (SD = 7.4), and almost all (98%) spoke English.	SoC-13	One-way ANOVAs were used to compare the groups, and associations between psychological distress and SoC were also assessed using hierarchical linear regression.	Used total scores: SoC-13 at baseline ranged from 16 to 90, with mean = 59.2 (SD = 15.0). SoC relatively stable between baseline and follow-up (*r* = 0.77, *P* < .001).	Higher baseline SoC-13 predicted lower distress at 12-month follow-up, and this pattern emerged irrespective of how distressed participants were at baseline. Beta = 0.27 *P* < .001.
Mahon et al.	To elucidate an extensive set of determinants of social anxiety among sexual minority individuals, by simultaneously examining experiences of discrimination and intraminority stress as predictors of social anxiety via various sexual minority-specific stressors and protective factors, and by a general psychological and social process (i.e. sense of coherence) found to predict social anxiety in the general population.	Adults expressing sexual minority identity. Excluded trans and non-binary.	Women *N* = 245, Men *N* = 256.	Age range (M ± SD): women 18–63 years (28.00 ± 9.66), men 18–62 years (30.29 ± 9.34). Sexual identity Gay/lesbian; women 42%, men 89%. Ethnicity white; women 96%, men 97%.	SoC-13 (range 13–91).	Correlations then Structural Equation Modelling to test hypothesized model using maximum likelihood estimation. Bootstrapping to test indirect effects	Men mean (SD) = 45.96 (14.59); Women mean (SD) = 48.34 (14.78).	SoC-13 Correlations: with Liebowitz Social Anxiety Scale = −0.55 (*P* < .01) (men) and −0.65 (*P* < .01) (women). With Brief Fear of Negative Evaluation Scale = −0.57 (*P* < .01) (men) and −0.59 (*P* < .01) (women). Internalized homonegativity −0.53 (*P* < .01) (men) and -0.50 (*P* < .01) (women). Experiences of discrimination −0.32 (*P* < .01) (men) and −0.15 (*P* < .05) (women). Intraminority stress −0.47 (*P* < .01) (men) and −0.43 (*P* < .01) (women). LGBTQ community connectedness 0.19 (*P* < .01) (men) and 0.01 (n-sig) (women). Indirect pathway from intraminority stress to social anxiety through SoC was significant for both women and men.
McDaid et al.	To examine how salutogenic, assets-based approaches to health improvement could function among GBMSM across diverse national contexts.	Gay and Bisexual men who have sex with men (GBMSM)	Data from two studies: SMMASH2: *N* = 2,971 overall and 1,715 for regression. Sex Now: *N* = 7,872(no SoC-13).	In SMMASH 2: 17% aged 16–25 years, 23% 26–35 years, 23% 35–45 years, and 37% 45 > years;. white 97% white.	SoC-13 in SMMASH 2 only	Cluster analysis used Observed/expected (O/E) ratio method. Chi-square and independent samples t-tests. Hierarchical logistic regression model.	No overall value given	SMMASH2: SoC-13 totals = 49.95 (11.16) for no syndemic ill health and 38.78 (13.3) for those with syndemic ill health. Chi-square = 15.492 (458.833), *P* < .001. Hierarchical logistic regression models: OR = 0.98 (95% CI 0.96–0.998). SoC-13 and emotional competence were sig associated with syndemic ill health in both bivariate correlations and significant predictor in logistic regression model.
McGarty et al.	To investigate: (i) The prevalence and association of depression and anxiety symptoms with sociodemographic, behavioural and stress factors, and (ii) The impact of minority stressors and resilience on depression and anxiety levels	Gay and Bisexual men, or those who sought sex with other men (GBMSM).	*N* = 3,077 in SMMASH2 study SoC analysis seems to involve *N* = 1,835 and 1,837.	Mean age was 39 years (range 16–78 years, SD = 13.5); 97.0% identified as white; 80.1% identified as gay; and 6.3% gender identity was transgender	SoC-13 (range is 0–78, indicating scored 0–6 instead of 1–7). In analysis range is stated as 0 to 13.	Binary logistic regression for sociodemographic variables/Hierarchical logistic regression—outcome was depression/anxiety Yes/No (based on cut-offs)	SoC-13 (Range 0–78): mean = 40.4 (SD = 13.4)	SoC-13 significant predictor of depression and anxiety at bivariate level OR = 0.86 (0.85–0.88), *P* < .001 (depression), OR = 0.87 (0.86–0.89), *P* < .001 (anxiety). Regression model OR = 0.85 (0.76–0.94), *P* < .001 (depression), OR = 0.89 (0.82–0.97), *P* = .01 (anxiety). Showed SoC-13 had a protective effect and significantly reduced the odds of moderate-to-severe depression and anxiety
Szymanski and Chung	‘It was hypothesized in this study that lesbian internalized heterosexism correlates negatively with sense of coherence’, ‘to examine the relationship between internalized heterosexism and self-identification as a feminist, attitudes toward feminism, involvement in feminist activities, and coping resources’	Lesbian and bisexual women aged 18 years or older.	*n* = 210	82% (*n* = 169) identified themselves as lesbian/gay, 17% (*n* = 36) as bisexual, and 1% (*n* = 2) as unsure. Ages ranged from 18 to 69 years, with a mean age of 38.02 years (SD = 10.84).	SoC-13 (7 point scale)	Hierarchical regression analysis	SoC-13 mean = 4.95 (SD = 0.93)	Hierarchical regression (controlling for age and income), identified attitudes toward feminism (*P* < .01), involvement in feminist activities (*P* < .01), and SoC-13 (*P* < .001) as unique predictors of internalized heterosexism (assessed using Lesbian Internalized Homophobia Scale).
SoC-9								
Issler et al.	Aim to replicate previous findings on the association between Childhood Gender Nonconforming, ACEs, and mental health problems in cisgender men. Additionally, our main aim is to research the moderating effects of sense of coherence in the association between ACEs and mental health problems.	Cis-gendered men	*n* = 371; Heterosexual *n* = 198; other sexual orientations *n* = 173	The mean age was 32.5 years (SD = 11.79; range: 18–69 years)	SoC-9 in German	Simple and moderated mediation models using the lavaan package	SoC-9: Overall, mean = 41.87 (SD = 10.40); Heterosexual men = 42.27 (SD = 10.32); Other sexual orientation *M* = 41.40 (SD = 10.5).	No significant difference in SoC-9 between the heterosexual and other sexual orientation groups. No significant difference for the moderator effect of SoC-9 on other measures, between heterosexual and men who have sex with men. Overall, among participants aversive childhood experiences were found to partly account for the relationship between childhood gender nonconformity and mental health in adulthood, with data indicating that sense of coherence could having a buffering effect on this link.
** **Other/Unclear								
Dyer	‘This study examined the intersection of religion, suicidality, and sexual orientation’.	Students aged under 30 years and with sexual orientation data	*n* = 20,991; Heterosexual *n* = 19,577; lesbian/gay *n* = 485; bisexual *n* = 696; and questioning *n* = 233.	37.0% (*n* = 7,757) male, 63.1% (13,234) female, with an average age of 22.5 years (SD = 3.3; range18–30).	Not clear how SoC was measured—“The sense of coherence scale measured the degree to which life is comprehensible, manageable, and meaningful.	Multistage	Unclear how measured, only use a as covariate.	Religiosity was significantly and positively associated ‘Coherence’ among heterosexuals, b(religiosity) = 0.02, 95% CI [0.02, 0.03] and bisexuals, b(religiosity) = 0.04, 95% CI [0.01, 0.08]. No association for lesbians/gay or questioning.
Veldorale-Griffin and Darling	How do transgender parents cope with and adapt to the disclosure of their gender transition (PGT)?	Transgender parents.	*N* = 73	Aged 26 to 68 years; 83% white. Birth gender: 72% male, 25% female, and 2.8% other; current gender 21% male, 74% female, and 5.6% other.	Nine items comprising the Comprehensibility and Manageability subscales of SoC (1 to 7 scale)	Hierarchical multiple regression and path analysis	Not reported	‘Findings suggested that stigma, boundary ambiguity, and sense of coherence had the greatest impact on family functioning and that a strong sense of coherence may act as a protective factor against the effects of stigma’.
Mixed method								
Kertzner	The study asked three questions: What is the subjective experience of getting older in a contemporary cohort of midlife gay men? What is the significance of homosexual identity and the HIV epidemic in self-appraisals of life experience associated with middle age? How do midlife gay men make sense of their personal histories and how might such appraisals be related to attitudes towards ageing and outlook towards the future?	Middle-aged gay men (aged 40–55 years)	*n* = 30	Average age = 45.6 ± 3.9 years.	Coherence assessed from qualitative analysis of text and rated on 3 point ranking. Three criteria based on Cohler’s (1982) were used for rating coherence in the narratives: the extent to which participants perceived a sense of meaning in their lives, experienced social validation of personal experience by others, and felt a sense of continuity between earlier and present life experiences.	Phenomenological approach used for interviews. Correlational analyses for quant measures.	NA	Quantitative: The three-point ranking of coherence was correlated with commitment to homo identity, worry about growing old, generativity and integrity. Relevant qualitative theme: Coherence and how much lives made sense to them.
**Qualitative**								
Bjorkman and Malterud	The study explored lesbian women’s successful coping experiences related to sexual minority stress.	Lesbian women.	*N* = 61 (64 histories given)	46% aged 18–29 years; 22% 30–39 years, 8% 40–49 years, 21% 50–59 years.	Question: ‘Describe your actions when you—in the role of a lesbian woman—successfully coped with a difficult experience—within or outside the healthcare system’.	Thematic analysis (supported by theories of stress and coping, and salutogenesis)	NA.	Two themes identified: ‘Disclosure and openness are means to counter anticipated prejudice’, and ‘maintaining dignity when prejudice appears’. Both themes stem from a salutogenic approach, i.e. focussing on successful coping strategies rather than on when things go wrong.
Fish, Williamson, and Brown	To explore the conditions under which a sample of British LGB cancer patients revealed their sexual orientation in hospital settings to enable a more nuanced approach to understanding disclosure in this context. RQs: What are the potential salutogenic factors that LGB cancer patients can draw on and how can this be enhanced in oncology care? How is disclosure a potentially salutogenic resource for better coping with cancer for LGB people?	LGB cancer patients.	*N* = 30.	Aged 24–77 years. All cisgender. 15 gay, 3 bisexual, 11 lesbian, 1 queer.	Semi-structured interview schedule, used flexibly.	Data were analysed using thematic analysis and interpreted and interrogated through salutogenesis theory ‘As the analysis developed we recognized that the concept of salutogenesis, defined by Jonas et al. as ‘the process of healing and health creation’ provided a useful and relevant lens with which to interrogate and interpret the data at the latter stages of theme development and refinement’.	NA	‘This study articulates the salutogenic potential of disclosure for LGB patients in enhancing quality of life and recovery’. Three themes were presented: ‘Authenticity as a driver for disclosure in cancer care’, ‘Partners as a (potential) salutogenic resource’ and ‘Creating safe, salutogenic healing environments conducive to disclosure’, Authors conclude that utilizing a salutogenic approach and establishing inclusive conditions that encourage disclosure can enhance the overall health of LGB individuals, regardless of whether they choose to disclose their identity.

### Measurement of sense of coherence in LGBTQ+ populations

Heterogeneity in the scoring of the sense of coherence measures in the quantitative studies and the diversity of populations studied severely limited comparison and prevented the use of meta-analytic techniques. Although the sense of coherence items are normally measured on a seven-point scale, not all studies did so (Table 3). Studies also differed in how they calculated the total scores—some summed the items (e.g. the SoC-29 scale would yield scores of 29 to 203), while others used the mean (e.g. the SoC-29 scale should yield scores of 1 to 7). Only one study, Kotowska et al. (2020), reported the scores for each of the three dimensions of sense of coherence (for the SoC-29 scale).

The most commonly used measure was SoC-13, however, four studies using this reported the total scores (Lyons et al. 2014, McDaid et al. 2020, Mahon et al. 2021, McGarty et al. 2021), and three reported mean scores (Szymanski and Chung 2003, Breidenstein et al. 2019, Ciria-Barreiro et al. 2021). Some comparison is possible for the SoC-13 total score in men who have sex with men as four studies reported the total score in this group (Lyons et al. 2014, McDaid et al. 2020, Mahon et al. 2021, McGarty et al. 2021). This score was lowest (mean = 40.4, standard deviation [(SD] = 13.4) for a broad sample recruited from the population of men who have sex with men in the United Kingdom and the Republic of Ireland (McGarty et al. 2021) and highest (mean = 59.2, SD = 15.0) for gay men aged over 40 years recruited in Australia (Lyons et al. 2014). Two studies with trans participants had used mean scores from the SoC-13 scale; these found sense of coherence was higher for German trans women aged over 18 years who had received gender-affirming surgery (mean = 4.97, SD = 1.11, (Breidenstein et al. 2019)) than among Spanish trans adolescents (mean = 3.69, SD = 1.00, (Ciria-Barreiro et al. 2021)). It is not possible to do any comparison for women who have sex with women as the two quantitative studies using the SoC-13 scale reported different scores: the mean score (Szymanski and Chung 2003) and the total score (Mahon et al. 2021), and the two studies with women who have sex with women using the SoC-29 scale also used either the mean score (Shechory Bitton and Noach 2022) or the total score (Kotowska et al. 2020).

### Comparing sense of coherence between population groups

Five studies compared sense of coherence values for participants from subpopulations of the LGBTQ+ community to sense of coherence values of participants who were heterosexual and/or cisgendered. Four studies found significantly lower sense of coherence scores among participants who were from LGBTQ+ communities (Table 3):—these compared cis-gender and transgender Spanish adolescents aged 15 to 18 years, (Ciria-Barreiro et al. 2021); Israeli Lesbian/Gay and heterosexual young adults aged 18 to 25 years, (Shechory Bitton and Noach 2022); Polish women aged 20 to 50 years who had sex exclusively or mostly with women and those who had sex exclusively with men (Kotowska et al. 2020); and Polish cisgender and non-cisgender, and heteronormative and non-heteronormative young adults aged 18 to 30 years (Jastrzębska and Błażek 2022). One study of cisgendered German men (Issler et al. 2023) found no difference (Table 3). A study of German trans women (Breidenstein et al. 2019), compared the sense of coherence score obtained with that for ‘normative samples reported in the respective test manuals’ and found no significant difference.

The study by Kotowska et al. (2020) of adult women in Poland also presented data for each of the three dimensions of sense of coherence (using the SoC-29 scale). It found women who had sex exclusively or mostly with women, when compared to women who had sex exclusively with men, scored lower on comprehensibility (*P* = .045) and manageability (*P* = .005) subscales. No difference was found between the two groups of women on the meaningfulness (*P* = .609) sub-scale (Table 3).

It was common for articles not to report sense of coherence separately for all groups, e.g. Ciria-Barreiro *et al*. (2021) or to only include it as a covariate, e.g. Dyer (2022). The study by Shechory Bitton and Noach (2022) conducted in Israel was the only one to report sense of coherence scores broken down by both gender and sexual orientation (SoC-29, using a 5-point scale), highest score was in heterosexual men (mean = 3.69, SD = 0.44), and lowest score in homosexual men (mean = 3.34, SD = 0.48).

### Associations between sense of coherence and measures of wellbeing, discrimination, and stigma

Sense of coherence was found to be associated with a range of other measures (Table 3). These included having a protective effect for moderate-to-severe depression and anxiety (McGarty et al. 2021) and psychological distress (Lyons et al. 2014); being associated with the presence of sexual, physical, or mental ill health outcomes (McDaid et al. 2020); and being negatively associated with both minority stress and social anxiety measures (Mahon et al. 2021), and psychological distress (Shechory Bitton and Noach 2022). A strong sense of coherence was identified as a possible protective factor against the effects of stigma in a sample of trans parents (Veldorale-Griffin and Darling 2016). Religiosity was associated with a higher sense of coherence in bisexual and heterosexual participants, but not in lesbians or gay men (Dyer 2022). Sense of coherence was positively associated with relationships with significant figures, but this result was not differentiated between LGBTQ+ and heterosexual participants (Shechory Bitton and Noach 2022).

### Qualitative exploration of salutogenesis

The two studies using qualitative approaches to explore salutogenesis (Table 3) were a study of lesbian, gay, and bisexual cancer patients in the UK (Fish et al. 2019) and a study of Norwegian lesbian women coping with difficult situations within or outside the healthcare system (Bjorkman and Malterud 2012). Both of these studies noted the salutogenic potential of disclosure of identity in relation to quality of life. The study of cancer patients identified three themes: ‘Authenticity as a driver for disclosure in cancer care’; ‘Partners as a (potential) salutogenic resource’; and ‘Creating safe, salutogenic healing environments conducive to disclosure’ (Fish et al. 2019). The study of lesbian women identified two themes: ‘Disclosure and openness are means to counter anticipated prejudice’; and ‘Maintaining dignity when prejudice appears’ (Bjorkman and Malterud 2012). Both studies suggest that disclosing sexual orientation and confidence in identity can benefit patient health. In the mixed methods study by Kertzner (1999), the application of sense of coherence or salutogenesis is ambiguous. The qualitative element of this study examined how middle-aged gay men made sense of ageing and look back on their lives, acknowledging the importance of life having meaning and their lives making sense to them. Although these concepts echo the salutogenic approach, the concept of salutogenesis is not explicitly discussed in this paper and they do not use a standard measure of sense of coherence in the quantitative analysis.

## DISCUSSION

This scoping review identified 17 studies that had explored salutogenesis, either qualitatively or through the measurement of sense of coherence, among LGBTQ+ populations. These studies had used a wide range of approaches and overall provided some evidence to indicate that a strong sense of coherence could buffer harms among LGBTQ+ populations. However, the studies included in this review varied greatly in their approach, and most had recruited comparatively small samples typically from with-in subgroups of the LGBTQ+ population, or samples recruited from specific contexts or from demographic subgroups in the wider population. Furthermore, all the studies included were from high-income countries, thus limiting the generalizability of the findings.

In the few studies that made this comparison, sense of coherence was often reported as being lower in the LGBTQ+ people studied than in the other population groups included (Kotowska et al. 2020, Ciria-Barreiro et al. 2021, Jastrzębska and Błażek 2022, Shechory Bitton and Noach 2022). There are several potential explanations for these findings. Sense of coherence is often considered to develop and stabilize in childhood and early adulthood (Antonovsky 1979, 1987, Super et al. 2016), and the populations recruited for these studies were younger in age. It may be, therefore, that the sense of coherence has not yet fully developed in these individuals. However, sense of coherence was lower than non-LGBTQ+ individuals of a similar age, suggesting further explanation is required. Two of these studies that reported a lower sense of coherence for LGBTQ+ participants were conducted in Poland (Kotowska et al. 2020, Jastrzębska and Błażek 2022) where acceptance of LGBTQ+ people is reportedly lower than in some other high-income countries (ILGA-Europe 2023, Our World in Data 2023, Equaldex 2024). Given sense of coherence’s conceptualization as comprehensibility, meaningfulness, and manageability, the life experiences of LGBTQ+ young people in these countries and elsewhere might either diminish or enhance the development of their sense of coherence. On the one hand, a feeling that one’s life is counter to the norms and expectations of one’s culture and society, exacerbated by experiences of stigma and discrimination and a lack of support from friends, family, and the wider community has the potential to curtail the development of sense of coherence in LGBTQ+ young people. However, being able to draw on other sources of support from LGBTQ+ networks or allies might enable coping, reduce the impact of stressors, and enhance sense of coherence. More research is needed on young LGBTQ+ people globally in order to understand the individual and sociocultural factors influencing the development of sense of coherence in this population. Indeed, this research should extend across the life course, as proponents of salutogenesis suggest that sense of coherence is continually shaped by life experiences, stressors, and individuals’ responses to these—suggesting targets for health promotion interventions to strengthen sense of coherence (Super et al. 2016, Langeland et al. 2022). Longitudinal studies would be of particular benefit in identifying changes in sense of coherence over time and the interactions between the three dimensions of sense of coherence and life experiences, stressors, and generalized resistance resources in LGBTQ + populations.

In line with previous research (Eriksson and Lindström 2006, 2007, Eriksson 2022), a few studies reported associations between sense of coherence and negative mental and physical health outcomes, and outcomes related to stigma and discrimination. All suggested a protective effect for sense of coherence, supporting the notion that sense of coherence is a health-promoting resource (Antonovsky 1979, 1987, 1996) in this population. Few studies explicitly reported associations or mediation analyses between sense of coherence and resources that might enhance coping, and none reported associations between the three dimensions of sense of coherence and any other factors. More research is therefore needed in order to identify the potential mediating effect of sense of coherence, and its three dimensions, between harms arising from external threats such as discrimination, stigma, or the impacts of public health emergencies on LGBTQ+ people.

Across the 14 studies that had measured sense of coherence quantitatively, this was most often measured using either the SoC-29 (4 studies) or SoC-13 (7 studies) scale. However, the results of these studies were presented in a range of different ways, including as the total score or as a mean score. Furthermore, several studies used rating scales to record participants’ responses to the sense of coherence items that varied from the seven-point scale used in the validated versions of these two measures (Eriksson and Mittelmark 2017). These issues severely limited our ability to make comparisons between studies. This review highlights the need for a standardized approach to the analysis and presentation of sense of coherence measures, or for studies that measure sense of coherence to report both the mean and total score.

The two qualitative studies (Bjorkman and Malterud 2012, Fish et al. 2019) that discussed salutogenesis suggest that when people feel confident and secure about their sexual orientation, and disclosing this, it can benefit their health. These findings suggest that healthcare professionals can contribute to physical and psychological wellbeing by providing conditions for lesbian, gay, and bisexual people to be their authentic selves. Doing so might enhance the three dimensions of sense of coherence by improving lesbian, gay, and bisexual people’s subjective experience of support, reducing feelings of disconnect from societal norms, and enabling them to live a life full of meaning. While synthesizing the findings between the qualitative and quantitative studies was challenging due to the varying conceptualisations and measurement of sense of coherence, a recent position paper on the future of salutogenesis encouraged alternative approaches to both, including qualitative research (Bauer et al. 2020).

### Strengths and limitations

This scoping review, through utilizing extensive searches, provides a comprehensive synthesis of the extant published peer-reviewed primary research evidence in relation to the examination of salutogenesis among LGBTQ+ populations, either qualitatively or through the measurement of sense of coherence. There are, however, several limitations to this review. Although we implemented an extensive search approach, it is possible that some relevant peer-reviewed studies were not identified through these. Furthermore, our review only included studies that were published in English and so omitted those published in other languages. We also restricted our inclusion to peer-reviewed publications, and so excluded grey literature. Further, due to heterogeneity of the included studies we did not undertake an assessment of publication bias, thus there could be bias if the results of any unpublished studies differed from those of the published studies. Combining quantitative and qualitative approaches to salutogenesis and sense of coherence does mean that concepts and definitions varied, making synthesis of findings challenging. Moreover, as this was a scoping review that considered a broad range of study types to assess the extent of current knowledge, we did not undertake a quality assessment. The included studies were undertaken with a diverse range of sub-populations, subgroups, and contexts so limiting the generalizability of the findings. Finally, the reviewed studies originated from high-income westernized countries, mainly in North America and Europe, consequently, the review findings will not be generalizable to all LGBTQ+ people, especially considering the heterogeneity of this population and varying sociocultural and legal contexts across the globe. Despite these limitations, this review illuminates the limited research in this area and highlights the inconsistency in reporting measures of sense of coherence as well as providing important insights for future research.

### Implications and future directions

This review suggests that both sense of coherence and salutogenic approaches to health in general are undervalued and under researched in LGBTQ+ populations. The potential role of sense of coherence in helping people to mobilize existing resources, including individual dispositional factors (such as personality and resilience), and external sociocultural factors (such as social support, education, and political stability), to help them cope with adversity is vital to promote and maintain good health and wellbeing (Antonovsky 1987). Simultaneously, developing and utilizing these resources to reduce the impact of stressors can either enhance or diminish one’s sense of coherence. Amongst LGBTQ+ people, feeling as if one’s life makes sense, has meaning, and is manageable, might enable them to understand and make use of resources that are available to them in order to prevent the negative impacts of stigma, discrimination, and health inequalities, and the potential amplification of these during public health emergencies—and further enhance their sense of coherence. In other populations, a strong sense of coherence has been found to mediate the harms arising from external threats, such as discrimination and stigma (Ying et al. 1997, Lam 2007, Switaj et al. 2013, Baron-Epel et al. 2017, Grothe et al. 2022) and to buffer the impacts of public health emergencies, such as the COVID-19 pandemic (Schäfer et al. 2020), on stress and mental health. Further research should therefore focus on the development, stability, and impact of sense of coherence in LGBTQ+ populations, with a view to developing public health interventions and messaging that might enhance sense of coherence and its effects. For example, interventions aiming to increase the skills and abilities of LGBTQ+ people and communities in understanding and perceiving life experiences and stressors in a way that enables them to identify appropriate generalized resistance resources might reduce their stress and other negative consequences and enhance their sense of coherence (Super et al. 2016, Bauer et al. 2020, Langeland et al. 2022).

### Conclusions

This review indicates that there has so far been limited research focussed on salutogenesis in LGBTQ+ populations. Most of the research conducted to date has involved the quantitative measurement of sense of coherence, using validated measures. This review found that the reporting of the data from these validated scales was variable and inconsistent across the studies and highlights a need for a standardized approach for reporting this data. The available, albeit very limited, evidence suggests that sense of coherence may be lower in some LGBTQ+ populations than in comparison groups of cis-gender heterosexuals, and that sense of coherence could help buffer the mental wellbeing of LGBTQ+ people from the effects of external threats such as stigma and discrimination, pandemics, and other emergencies. However, these need further investigation through robust studies that include sufficient numbers of participants from across the range of LGBTQ+ population subgroups. Studies exploring salutogenesis and sense of coherence and the potential of this to buffer mental wellbeing also need to be conducted across different sociocultural and legal contexts to reflect global differences in legislation, attitudes, and consequent LGBTQ+ experiences including levels of discrimination, prejudice, and victimization.

## Supplementary Material

daaf049_suppl_Supplementary_Material

## Data Availability

Data sharing is not applicable to this article as no new data were created or analysed in this study.
