# Supplementary material for: A scoping review of sense of coherence and salutogenesis among LGBTQ+ populations
Source: Health Promot Int. 2025 Apr 23;40(2):daaf049. doi: 10.1093/heapro/daaf049 (PMC12015610; doi:10.1093/heapro/daaf049)
Supplement: daaf049_suppl_Supplementary_Material [file daaf049_suppl_supplementary_material.docx]

**Online material**

**Annex 1**

**LGBTQ+ Sense of Coherence & Salutogenesis review: Review Plan**

***Review Type*:**

Scoping.

***Framework:***

Population, Concept, and Context (PCC) framework.

***Research question:***

To what extent has salutogenesis, either qualitatively or through measurement of sense of coherence, been explored among LGBTQ+ populations globally?

***Inclusion / Exclusion Criteria***

|  | ***Inclusion*** | ***Exclusion*** |
| --- | --- | --- |
| **PCC Components** |  |  |
| ***Population*** | Lesbian, gay, bisexual, trans, queer (LGBTQ+) people and communities, and associated terms (such as gender minorities, MSM, etc.), including studies of component populations, and where part of a general population study the data is reported separately for LGBTQ+ people | Where data is not reported for LGBTQ+ people, or any of the subgroups within this umbrella category. |
|  | Adults (all, or mostly, aged 18 years and over) | Only people aged under 18 years. |
| ***Concept*** | Quantitative measure of Sense of Coherence OR qualitative assessment of salutogenesis or Sense of Coherence | No Quantitative measure of Sense of Coherence OR qualitative assessment of salutogenesis or Sense of Coherence |
| ***Context*** | All Countries | - |
| **Limits** |  |  |
| ***Language*** | In English | Languages other than English |
| ***Study types*** | Primary studies: Quantitative, qualitative, or mixed methods. | Reviews |
|  |  | Case reports and series |
|  |  | Conference abstracts |
|  |  | Thesis |
|  |  | Commentaries & editorials |

***Core Search Terms/Strings:***

*Population:*

transgender* OR transsexual* OR "gender nonconforming" OR "gender identity disorder" OR "gender dysphoria" OR "gender minority" OR lesbian* OR gay* OR bisexual* OR "sexual minority" OR "same-sex" OR homosexual* OR “homosexuality, male” OR “homosexuality, female” OR “gender identity” OR non-heterosexual* OR “non heterosexual*” OR homosexuality OR queer* OR questioning OR "non-binary" OR "non binary" OR “LGBT*” OR “sexual dissident*” OR “sexual and gender minorities” OR “gender variant” OR gender-variant OR genderqueer OR intersex OR “minority groups” OR “TGNC” OR “transgender and gender nonconforming”

*Concept:*

Salutogenesis OR "Sense of Coherence"

***Databases:***

The databases to be searched are:

Medline All, PsycINFO, CINAHL plus (via EBSCOHost), Web of Science Core Content, and Scopus.

***Screening process:***

Screening will be undertaken by two people independently.

Title an abstract screening outcomes:-

Include + Include = Include in full text review
Include + maybe = include in full text review
Maybe + maybe = 3^rd^ person
Maybe + exclude = 3^rd^ person
Exclude + exclude = exclude

Full-text screening outcomes:-

Include + Include = Include in full text review
Include + maybe = include in full text review
Maybe + maybe = screening team
Maybe + exclude = screening team
Exclude + exclude = exclude

***Quality assessment:***

None as a scoping review.

***Data extraction / Charting:***

This will be undertaken by one team member and checked by another team member.

The following will be extracted:

Paper title

Author/s

Publication year

Country & Year of data collection

Study aim

Population/subgroups

Setting

Overall sample/subgroup size

Study methodology

Instruments used/Study procedure

Analysis method/s

Main findings

Findings related to Sense of Coherence & Salutogenesis

***Synthesis:***

The data will be synthesised as a descriptive narrative.

**Annex 2**

**Preferred Reporting Items for Systematic reviews and Meta-Analyses extension for Scoping Reviews (PRISMA-ScR) Checklist**

Page numbers relate to the submitted version.

| **SECTION** | **ITEM** | **PRISMA-ScR CHECKLIST ITEM** | **REPORTED ON PAGE #** |
| --- | --- | --- | --- |
| **TITLE** | | | |
| Title | 1 | Identify the report as a scoping review. | 1, 2 |
| **ABSTRACT** | | | |
| Structured summary | 2 | Provide a structured summary that includes (as applicable): background, objectives, eligibility criteria, sources of evidence, charting methods, results, and conclusions that relate to the review questions and objectives. | 2 |
| **INTRODUCTION** | | | |
| Rationale | 3 | Describe the rationale for the review in the context of what is already known. Explain why the review questions/objectives lend themselves to a scoping review approach. | 3-5 |
| Objectives | 4 | Provide an explicit statement of the questions and objectives being addressed with reference to their key elements (e.g., population or participants, concepts, and context) or other relevant key elements used to conceptualize the review questions and/or objectives. | 5, 6 |
| **METHODS** | | | |
| Protocol and registration | 5 | Indicate whether a review protocol exists; state if and where it can be accessed (e.g., a Web address); and if available, provide registration information, including the registration number. | 6, Annex 1 |
| Eligibility criteria | 6 | Specify characteristics of the sources of evidence used as eligibility criteria (e.g., years considered, language, and publication status), and provide a rationale. | 6, Table 1 |
| Information sources* | 7 | Describe all information sources in the search (e.g., databases with dates of coverage and contact with authors to identify additional sources), as well as the date the most recent search was executed. | 6 |
| Search | 8 | Present the full electronic search strategy for at least 1 database, including any limits used, such that it could be repeated. | Annex 1, Annex 3 |
| Selection of sources of evidence† | 9 | State the process for selecting sources of evidence (i.e., screening and eligibility) included in the scoping review. | 6-7 |
| Data charting process‡ | 10 | Describe the methods of charting data from the included sources of evidence (e.g., calibrated forms or forms that have been tested by the team before their use, and whether data charting was done independently or in duplicate) and any processes for obtaining and confirming data from investigators. | 7 |
| Data items | 11 | List and define all variables for which data were sought and any assumptions and simplifications made. | 7 |
| Critical appraisal of individual sources of evidence§ | 12 | If done, provide a rationale for conducting a critical appraisal of included sources of evidence; describe the methods used and how this information was used in any data synthesis (if appropriate). | N/A |
| Synthesis of results | 13 | Describe the methods of handling and summarizing the data that were charted. | 7 |
| **RESULTS** | | | |
| Selection of sources of evidence | 14 | Give numbers of sources of evidence screened, assessed for eligibility, and included in the review, with reasons for exclusions at each stage, ideally using a flow diagram. | 8, Figure 1 |
| Characteristics of sources of evidence | 15 | For each source of evidence, present characteristics for which data were charted and provide the citations. | 8-9 |
| Critical appraisal within sources of evidence | 16 | If done, present data on critical appraisal of included sources of evidence (see item 12). | N/A |
| Results of individual sources of evidence | 17 | For each included source of evidence, present the relevant data that were charted that relate to the review questions and objectives. | Table 2, Table 3, 9-11 |
| Synthesis of results | 18 | Summarize and/or present the charting results as they relate to the review questions and objectives. | 8-11 |
| **DISCUSSION** | | | |
| Summary of evidence | 19 | Summarize the main results (including an overview of concepts, themes, and types of evidence available), link to the review questions and objectives, and consider the relevance to key groups. | 12 |
| Limitations | 20 | Discuss the limitations of the scoping review process. | 13-14 |
| Conclusions | 21 | Provide a general interpretation of the results with respect to the review questions and objectives, as well as potential implications and/or next steps. | 14-15 |
| **FUNDING** | | | |
| Funding | 22 | Describe sources of funding for the included sources of evidence, as well as sources of funding for the scoping review. Describe the role of the funders of the scoping review. | 16 |

JBI = Joanna Briggs Institute; PRISMA-ScR = Preferred Reporting Items for Systematic reviews and Meta-Analyses extension for Scoping Reviews.

* Where *sources of evidence* (see second footnote) are compiled from, such as bibliographic databases, social media platforms, and Web sites.

† A more inclusive/heterogeneous term used to account for the different types of evidence or data sources (e.g., quantitative and/or qualitative research, expert opinion, and policy documents) that may be eligible in a scoping review as opposed to only studies. This is not to be confused with *information sources* (see first footnote).

‡ The frameworks by Arksey and O’Malley (6) and Levac and colleagues (7) and the JBI guidance (4, 5) refer to the process of data extraction in a scoping review as data charting*.*

§ The process of systematically examining research evidence to assess its validity, results, and relevance before using it to inform a decision. This term is used for items 12 and 19 instead of "risk of bias" (which is more applicable to systematic reviews of interventions) to include and acknowledge the various sources of evidence that may be used in a scoping review (e.g., quantitative and/or qualitative research, expert opinion, and policy document).

*From:* Tricco AC, Lillie E, Zarin W, O'Brien KK, Colquhoun H, Levac D, et al. PRISMA Extension for Scoping Reviews (PRISMAScR): Checklist and Explanation. Ann Intern Med. 2018;169:467–473. [doi: 10.7326/M18-0850](http://annals.org/aim/fullarticle/2700389/prisma-extension-scoping-reviews-prisma-scr-checklist-explanation).

**Annex 3**

**MEDLINE searches**

1: TS=((transgender* OR transsexual* OR "gender nonconforming" OR "gender identity disorder" OR "gender dysphoria" OR "gender minority" OR lesbian* OR gay* OR bisexual* OR "sexual minority" OR "same-sex" OR homosexual* OR “homosexuality, male” OR “homosexuality, female” OR “gender identity” OR non-heterosexual* OR “non heterosexual*” OR homosexuality OR queer* OR questioning OR "non-binary" OR "non binary" OR “LGBT*” OR “sexual dissident*” OR “sexual and gender minorities” OR “gender variant” OR gender-variant OR genderqueer OR intersex OR “minority groups” OR “TGNC” OR “transgender and gender nonconforming”))

2: MHX=("sexual and gender minorities"/ OR Homosexuality/ OR Homosexuality, Female/ OR Homosexuality, Male/ OR Bisexuality/ OR Transgender Persons/)

3: #2 OR #1

4: TS=(Salutogenesis OR "Sense of Coherence")

5: MHX=(Sense of Coherence)

6: #4 OR #5

7: #3 AND #6
